# Multi-Scale Modeling of Transport Properties in Cementitious Materials with GO Admixture

**DOI:** 10.3390/nano15030222

**Published:** 2025-01-30

**Authors:** Bing Liu, Weichen Kang, Weixing Lian, Feng Xing, Hongfang Sun, Hongyan Ma

**Affiliations:** 1 School of Traffic and Environment, Shenzhen Institute of Information Technology, Shenzhen 518172, China; 2 Guangdong Provincial Key Laboratory of Durability for Marine Civil Engineering, College of Civil and Transportation Engineering, Shenzhen University, Shenzhen 518060, China; 3 Department of Civil, Architectural, and Environmental Engineering, Missouri University of Science and Technology, Rolla, MO 65409, USA

**Keywords:** GO, multi-scale modeling, transport property, microstructure model, ITZ modeling

## Abstract

In coastal areas, the presence of concrete cracks provides pathways for hazardous ions to ingress from the exterior into the interior of concrete, while the migration of the ions further accelerates concrete deterioration and causes durability problems. The incorporation of graphene oxide (GO) into concrete can inhibit crack initiation and development starting at the nanoscale, improving the concrete microstructure, thereby affecting concrete’s resistance to hazardous ion transport and the resulting deterioration. In this study, a multi-scale transport model for cementitious materials with a GO admixture was established to predict the resistance to hazardous ions. Based on the determination of hydration types and hydration kinetics, microstructure modeling was conducted at three scales, the sub-microscale, microscale, and mesoscale, upon which transport property simulations were performed. At the microscale, the effects of both the cement paste matrix and the interfacial transition zone (ITZ) were considered. Through the simulation, it was found that the addition of GO reduced the duration of the induction period and increased the rate of hydration development after the induction period. Moreover, the incorporation of GO could reduce the porosity of cementitious materials at all simulation scales at both early and later ages. At the microscale, it improved the pore structure of the cement matrix and ITZ by reducing large pores and increasing small pores. At all three simulation scales, GO could increase the diffusion tortuosity in hydration products, suppress ion transport, and improve the resistance to hazardous ions of cementitious materials.

## 1. Introduction

The durability of concrete structures plays a crucial role in their long-term performance, especially when exposed to severe environmental conditions [1]. The durability of concrete is predominantly governed by its transport properties [2]. Actually, the movement of water and ions through the nano- and micron-sized pore network in hardened cement paste significantly influences concrete’s performance, such as resistance to permeability and erosion, creep response, and dimensional stability. In marine environments, for instance, the penetration of aggressive ions such as chlorides into cementitious materials can initiate detrimental degradation processes, notably reinforcement corrosion, leading to structural deterioration and a reduced service life [3]. Thus, understanding the transport properties of these materials is crucial for ensuring long-term structural performance.

The incorporation of nanomaterials has been considered as a promising approach to enhance the transport properties of cementitious materials. Various nanomaterials have been investigated, such as nano-SiO_2_ [4,5], nano-TiO_2_ [6,7,8], nano-CaCO_3_ [9,10], carbon nanotubes (CNTs) [11,12], carbon nano-fibers (CNFs) [13,14], etc. Among these nanomaterials, graphene oxide (GO) has emerged as an outstanding nano-strengthening material, attracting sustained attention from both academic and industrial communities owing to its exceptional properties, including a high specific surface area (700–1500 m^2^/g), superior elastic modulus (23–42 GPa), and remarkable tensile strength (130 GPa) [15]. Compared with reduced GO or pure graphene, GO facilitates practical cementitious material mixing due to the presence of functional groups such as hydroxyl and epoxide groups within its structure [2]. Furthermore, these functional groups can react with hydration products and transform the weak van der Waals forces between nanomaterials and cement paste into robust chemical bonds [2,16]. Additionally, GO provides nucleation sites for early cement hydration and functions as a physical filler, thereby modifying the concrete microstructure and enhancing its resistance to both hazardous ionic transport and subsequent material deterioration [17].

Recent experimental studies have demonstrated the significant impact of GO on the properties of cementitious materials. Mercury intrusion porosimetry (MIP) tests conducted by Gong et al. [18] revealed that GO not only reduces the total porosity but also decreases the number of capillary pores. Similarly, research by Mohammed et al. [2] established that small dosages of GO can effectively inhibit chloride ion penetration and reduce water absorption through pore structure refinement. Notably, with a GO content of merely 0.01%, the penetration depth of chloride ions in cementitious materials was substantially reduced from 26 mm to 5 mm. Additionally, GO enhances the strength properties of cementitious materials due to its superior tensile properties compared to 0D nanomaterials and its ability to inhibit crack initiation at the nanoscale [19]. Within the GO dosage range of 0.01–0.04% by mass of cement, the chloride migration coefficient and flexural strength reached their minimum and maximum values, respectively, at 0.03% GO content, suggesting the existence of an optimal dosage of GO for enhancing cementitious material properties. If the optimal value could be determined through numerical simulation, it could effectively reduce the experimental time and iterations while improving the precision of optimization.

Therefore, although investigations of GO’s transport properties have been conducted with experimental approaches, these tests face significant challenges. Experimental testing is time-consuming, and the resulting data often exhibit considerable variability due to differences in measurement techniques and sample preparation procedures [3]. To address these limitations, numerical simulations offer an alternative approach for predicting and analyzing transport properties. However, current simulation studies examining the interaction between GO and cementitious materials are rare, primarily focusing on molecular dynamics simulations of the interface strength between GO functional groups and calcium silicate hydrate (C-S-H). For instance, Hou et al. [20] employed molecular dynamics simulations alongside experimental studies to investigate the effects of GO on the cement paste hydration, microstructure, and mechanical properties, elucidating the interaction mechanisms between graphene and cement hydration products. Alkhateb et al. [21] utilized molecular dynamics to evaluate the interface strength between calcium silicate hydrate and functionalized graphene nanosheets. Fan et al. [22] simulated interfacial stress transfer mechanisms in GO-reinforced cement composite systems, validating their findings through pullout tests. Nevertheless, comprehensive simulation studies examining ion transport in GO-modified cementitious materials remain scarce.

To address this research gap, this study aimed to conduct multi-scale simulations of transport properties in GO-modified cementitious materials through the development of hydration kinetics models, microstructure models, and random walker algorithms. This comprehensive approach revealed the influence of GO on the microstructure of cementitious materials and characterized the transport behavior of ions within the modified matrix. The implementation of a multi-scale simulation methodology ensured an optimal balance between computational efficiency and numerical accuracy across different modeling scales.

## 2. Research Significance

A quantitative model is needed to predict the transport properties of GO-modified cementitious materials. The multi-scale modeling approach adopted in this study enables rapid and accurate bridging between sub-microscopic-, microscopic-, and mesoscopic-scale properties. The model development integrated hydration kinetics, microstructural evolution, and random walk algorithms at sequential hierarchical stages. This comprehensive framework facilitates both the understanding of the influence mechanisms of GO on cementitious materials and the prediction of ionic transport behavior within the modified matrix.

## 3. Materials and Methods

### 3.1. Materials

The cement used in this work was produced by Aosaier Technology Co., Ltd. (Fushun, China), with the composition shown in Table 1. The OPC mentioned later refers to this cement.

The sand used was produced by ISO Standard Sand Co., Ltd. (Xiamen, China). Before incorporating it into mortar specimens, sand particles larger than 1 mm in diameter were sieved out, as the presence of large sand particles would cause greater discreteness in the properties of mortar specimens [23,24].

The GO admixture used in this study was prepared in the laboratory using an electrochemical method. In this method, carbon fiber cloth and a stainless steel plate were used as the anode and cathode, respectively, with tap water serving as the electrolyte solution. A current of 10 mA was applied for 5 days to produce the GO suspension. The detailed preparation procedure and characterization of GO can be referred to in Ref. [25].

The preparation process for GO-modified cement paste was as follows. GO was first dispersed in the deionized water used for the cement paste and sonicated for 5 min. Then, it was added to OPC at a replacement level of 0.1% by weight of the OPC. Subsequently, the mixture was mixed at a water-to-binder ratio of 0.45, poured into 10 mm × 10 mm × 40 mm molds, and consolidated. Then, the samples were demolded after 24 h and cured until the required ages (1 day, 3 days, 7 days, 14 days, 28 days, 56 days) for further testing. The preparation of GO-modified cement mortar specimens was essentially the same as that of the cement paste specimens, except that sand was added during mixing with a cement-to-sand ratio of 1:1. The preparation of a control paste and mortar followed the same process but without the addition of a GO admixture.

### 3.2. Testing

DTG-TGA testing was performed to identify the composition of both OPC and OPC-GO pastes and to calculate the degree of hydration at varied ages and thereby establish hydration kinetics. The testing was conducted using a TGA/DSC3+ thermal analyzer produced by Mettler Toledo, with a temperature range from room temperature to 1000 °C, heating rate of 10 °C/min, and flow rate of 50 mL/min of nitrogen. The heating process consisted of two steps: first, heating the paste from room temperature to 105 °C and maintaining the temperature at 105 °C for 10 min to ensure the complete removal of free water through evaporation, then heating the powders from 105 °C to 1000 °C at a rate of 10 °C/min. The detailed testing procedures and data analysis are described in Ref. [26]. Although drying at 105 °C may lead to an underestimation of the hydrate water content and, consequently, the degree of hydration, this methodology aligns with the recommendations of the National Institute of Standards and Technology (NIST) [26]. This approach provides sufficient reliability for the comparative assessment of hydration degrees between OPC and OPC-GO specimens, though it may not be suitable for the absolute quantification of hydration degrees.

BJH was used to determine the gel pore distribution (3 nm~200 nm) in both OPC and OPC-GO pastes at different ages. Combined with the volume density obtained from mercury intrusion porosimetry (MIP), this allowed for the calculation of the volume fraction of gel pores in the cement paste. This volume fraction could then be converted to the volume fraction of gel pores in the outer C-S-H layer, enabling the establishment of a model for the outer C-S-H layer. The equipment for this test used an ASAP 2460 specific surface area and pore size analyzer (Micromeritics, Norcross, USA). Prior to testing, the samples underwent high-temperature (200 °C) nitrogen degassing treatment for 2 h to remove excess gas from the samples, after which they were loaded into the instrument for testing.

MIP testing was conducted to obtain the volume density of the cement paste. Combined with the BJH results, this would allow for the calculation of the volume fraction of gel pores in the cement paste. The testing was conducted using an AutoPore IV 9500 mercury porosimeter (Micromeritics, Norcross, USA) with an accuracy of ±1% and a test pressure range of 0.10–30,000 psia.

SEM-BSE was used to investigate the conversion ratio of low-density (LD) C-S-H to high-density (HD) C-S-H and to accurately establish microstructural computer models for cement paste with GO. Additionally, the accuracy of the models was also verified through porosity measurements with SEM-BSE. Before capturing BSE images, specimens had to undergo a series of sample preparation procedures. First, a low-speed saw was used to cut the specimens into 10cm^3^ cubes. Epoxy resin was then injected into the samples to stabilize their microstructure. Next, the epoxy-injected sample surfaces were polished using a polishing machine with diamond polishing pastes down to 0.15 µm to achieve a smooth surface. Finally, the samples were gold-coated to form a conductivity surface and loaded into an SEM chamber. The SEM used a Phenom ProX scanning electron microscope (FEI Company, Eindhoven, the Netherlands) operating at a 15 kV voltage. The magnification of the image was 800× with an image resolution of 1536 × 1103. For each specimen, 30 BSE images were taken of different areas for analysis. The porosity analysis was based on the grayscale threshold method [23,27,28,29,30]. The distinction made between LD C-S-H and HD C-S-H was based on the morphological characteristics of these two components in the BSE images [23,24]. The LD C-S-H is the internal layer of C-S-H, typically located adjacent to unhydrated cement particles. In BSE images, it appears smooth and homogeneous. Due to its dense microstructure, LD C-S-H remains largely impervious to external ionic penetration, making it a non-transportable phase for ions such as chloride (Cl^−^). In contrast, the HD C-S-H is the external layer of C-S-H and exhibits higher porosity in BSE images. The presence of these pores facilitates ionic diffusion, making HD C-S-H a transportable phase for ions like Cl^−^. This distinction between LD C-S-H and HD C-S-H is crucial for understanding the transformation of low-density C-S-H to high-density C-S-H following GO admixture incorporation.

## 4. Results

### 4.1. Hydration Study of Cementitious Materials with GO

#### 4.1.1. Reaction of OPC with GO Admixture

For the OPC-GO system, it was reported that the addition of GO affected the hydration products of cement [31,32]. In the system, GO acted as a nucleation site for hydration products, which caused part of the LD C-S-H to transform into HD C-S-H due to aggregation effects, thereby altering the composition of cement hydration products.

However, when researchers used chemical equations to investigate cement hydration reactions and establish hydration models for cementitious materials, they generally did not distinguish between the two forms of C-S-H gel (LD C-S-H and HD C-S-H) in terms of their chemical formulas, and the simulation results proved to be reasonable [33]. Therefore, in our model, LD and HD C-S-H are also not distinguished for the hydration reaction. The influence of the transformation of LD C-S-H to HD C-S-H on the microstructure model of cement paste will be considered in Section 4.2.2.

On this basis, when establishing a hydration model for the OPC-GO system, the methodology for the OPC-GO system can be similar to that of the OPC system, as the types of hydration reactions are no different from those in the OPC system. Herein, we adopt the equations proposed by Bentz et al. [34,35] to describe the hydration reactions of OPC, as these have been successfully used to explain the behaviors of cement-based materials and have been validated for their rationality. The volume change of the cementitious materials after hydration can be found in Ref. [36], which was used for establishing the microstructure model of cement paste in Section 4.2.

#### 4.1.2. Hydration Kinetics of OPC-GO System

Based on the hydration reaction that occurred in the OPC-GO system, the hydration kinetics model of the OPC-GO system should be similar to that of the OPC system. In both systems, a three-parameter exponential degree of hydration can be used to model the hydration development [37], as shown in Equation (1).

(1) $$\alpha (t)=\alpha_{u}\cdot e^{-{\left(\frac{\tau }{24\cdot t}\right)}^{\beta }}$$

where α(t) is the hydration level at the time t. $\alpha_{u}$, τ, and β,respectively, represent the ultimate degree of cement hydration, the duration of the induction period, and the rate of hydration after the induction period ends.

**Figure 1:**
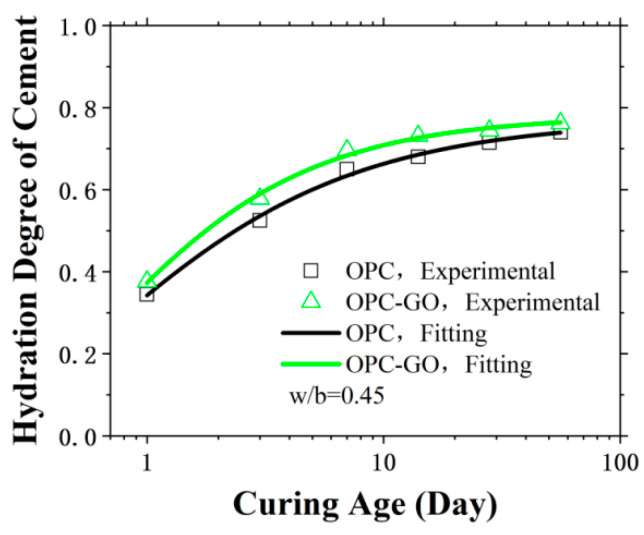
Degree of cement hydration and hydration kinetics model fitting results for OPC and OPC-GO composite systems.

In order to obtain the values for the coefficients $\alpha_{u}$, τ, and β, TGA experiments were first conducted on cement paste for both OPC and OPC-GO systems. Based on the TGA results, the degree of hydration of OPC can be calculated using Equation (2) [26,38,39,40,41].

(2) $$\alpha =W_{w}/W_{t}\times 100\%$$

where W_w_ represents the mass of chemically bound water lost in OPC at the time t; W_t_ represents the total mass of chemically bounded water lost in fully hydrated OPC. The test results can be seen in Figure 1. As shown in the figure, the addition of GO can accelerate the hydration process of cement-based materials and increase the degree of hydration at all ages. Specifically, at the four curing ages of 1 day, 3 days, 7 days, and 14 days, the degree of hydration of OPC in the OPC-GO system increased by more than 7% compared to that of the OPC system, reaching approximately 10% on the third day.

Then, Equation (1) was used to fit the TGA test results of both the OPC and OPC-GO systems, obtaining the coefficients and corresponding hydration kinetics models for the two systems. The results are shown in Figure 1 and Table 2. According to the kinetic parameters obtained from the fitting, the addition of GO reduced the duration of the induction period and increased the rate of hydration development after the induction period ended. This was mainly because there are hydrophilic oxygen-containing functional groups on the surface of GO, such as hydroxyl, carbonyl, and carboxyl groups. During the cement hydration process, these functional groups attract nearby water molecules, causing cement hydration products like C-S-H gel to use GO as a crystal nucleus for growth, attaching to and precipitating on the GO surface [37]. This nucleation effect of GO not only accelerates the early hydration of cement but also increases the ultimate degree of cement hydration.

With the hydration kinetics models, the degree of hydration of both OPC and OPC-GO systems can be calculated at any hydration time, t.

### 4.2. Modeling of Multi-Scale Microstructure of Cementitious Materials with GO

For cementitious materials, the microstructural characteristics are manifested across multiple scales, ranging from the structure of C-S-H gel (10^−10^~10^−9^ m) to that of cement mortar (>10^−2^ m), crossing multiple orders of magnitude. Therefore, it was necessary to establish a computer model of the microstructure at multiple scales for transportable ions to obtain a more precise simulation result [42,43,44,45].

At the sub-microscale (10^−9^~10^−6^m), there exist unhydrated cement, an inner hydration product layer, an outer hydration product layer, and capillary pores, which together form the cement paste. Through the arranging of these phases in an assumed cement paste room, the capillary pores can be formed and quantified. Among these phases, unhydrated cement is a non-transportable phase for ions, while capillary pores are transportable phases. As for the inner and outer hydration product layers, the inner hydration product layer consists of HD C-S-H and nanoscale crystals, while the outer hydration layer is composed of outer crystalline phases (CH, AFt, AFm, etc.) at the sub-microscale and an outer C-S-H layer consisting of LD C-S-H and gel pores. In both the inner and outer hydration layers, crystalline phases and C-S-H gel phases account for 40% and 60% of the phases, respectively [33]. The crystalline phases are considered non-transportable, while the outer C-S-H layer is transportable. Since HD C-S-H has a lower gel porosity compared to LD C-S-H, this results in HD C-S-H having a transport coefficient approximately two orders of magnitude lower than that of LD C-S-H [33], which can be approximated as zero. Therefore, at the sub-microscale, only the microstructure of the outer C-S-H layer should be modeled.

At the microscale (10^−6^~10^−4^ m), the mortar needs to be divided into two parts due to the presence of an interfacial transition zone (ITZ) around aggregate particles. This is the weakest part of the cement mortar, exhibiting higher porosity compared to the cement matrix, so the transport properties of the two parts may differ. To ensure the accuracy of transport simulations, the ITZ should be modeled separately and distinguished from the cement matrix.

At the mesoscale (10^−4^~10^−2^ m), herein, only a mortar model was considered instead of a concrete model since the existence of coarse aggregates in concrete requires excessive running memory and REV space during simulation, which cannot be implemented on a personal computer. Therefore, at this scale, the only transportable phase that needed to be modeled was the cement mortar.

#### 4.2.1. The Microstructure Modeling of the Outer C-S-H Layer at the Sub-Microscale

To establish the microstructure models of the outer C-S-H layer for OPC and OPC-GO composite systems, a self-similar aggregation algorithm [18] was adopted. This algorithm has been applied in previous research, and the rationality and accuracy of the established microstructure models have been validated [18]. In the algorithm, the required input parameter is the porosity of gel pores in the outer C-S-H layer, which controls the termination of the modeling process and generates the outer C-S-H layer microstructure. This parameter could be obtained through the following experiments [33]. First, the gel pore size distribution was obtained through BJH experiments. Then, using MIP experiments, the volume density was obtained, from which the porosity of gel pores in cement paste could be calculated. The porosity of gel pores in OPC and an OPC-GO system is shown in Table 3.

Since gel pores are located in the outer hydration layer, the porosity of gel pores in the outer hydration layer was calculated according to Equation (3).

(3) $$\varphi_{\mathrm{SCP}}^{\mathrm{OL}}=\frac{\varphi_{\mathrm{SCP}}^{P}}{\varphi_{\mathrm{SCP}}^{P}+\frac{(k_{v}^{\mathrm{cem}}-1)\alpha_{\mathrm{cem}}}{1+\rho_{\mathrm{cem}}w/c}}$$

where $\varphi_{\mathrm{SCP}}^{\mathrm{OL}}$ represents the porosity of gel pores in the outer hydration layer, while $\varphi_{\mathrm{SCP}}^{P}$ represents the porosity of gel pores in the cement paste. $k_{v}^{\mathrm{cem}},\;\alpha_{\mathrm{cem}},and\rho_{\mathrm{cem}}$ represent the total volume of hydration products, the cement hydration degree, and the cement density, respectively.

The outer hydration layer consists of the outer C-S-H layer and outer crystalline phases, with fractions of 60% and 40%, respectively. Therefore, the porosity of gel pores in the outer C-S-H layer was calculated according to Equation (4).

(4) $$\varphi_{\mathrm{SCP}}^{\mathrm{OCSHL}}=\frac{\varphi_{\mathrm{SCP}}^{\mathrm{OL}}}{0.6+0.4\varphi_{\mathrm{SCP}}^{\mathrm{OL}}}$$

**Figure 2:**
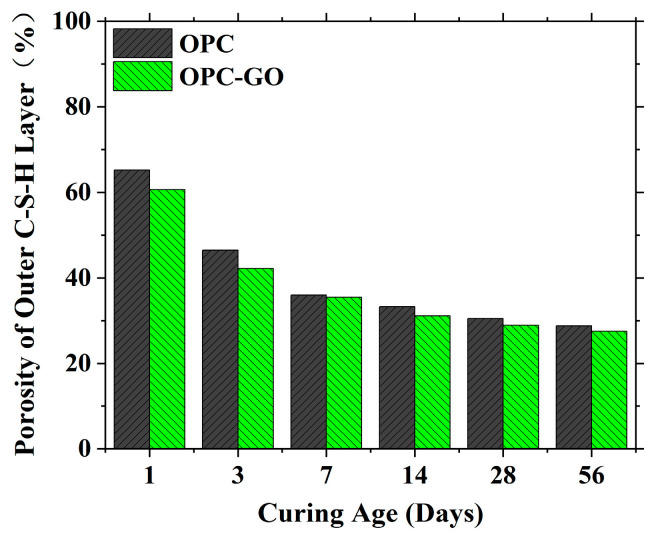
The porosity of gel pores in the outer C-S-H layer $\left(\varphi_{SCP}^{OCSHL}\right)$ in OPC and OPC-GO systems.

Figure 2 shows the obtained porosity of the outer C-S-H layer for OPC and OPC-GO systems at varied ages. As can be seen, whether in early or late hydration stages, the addition of GO can significantly reduce the porosity of the outer C-S-H layer.

**Figure 3:**
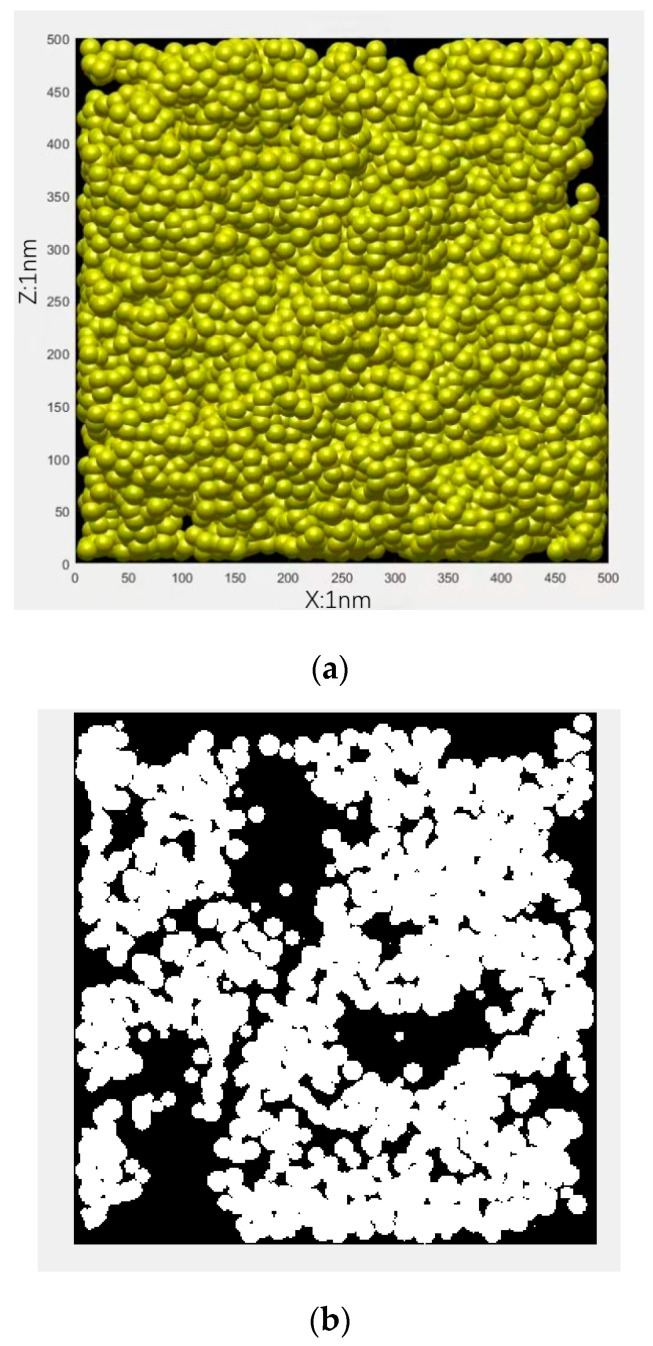
Microstructure of the outer C-S-H layer. (**a**) 3D; (**b**) 2D.

Based on the obtained porosity of gel pores in the outer C-S-H layer and using the self-similar aggregation algorithm, the outer C-S-H layer microstructure model could be established and is shown in Figure 3.

#### 4.2.2. Microstructure Modeling of Bulk Cement Paste in Mortar at Microscale

Cement paste consists of unhydrated cement, inner hydration products, outer hydration products, and large capillary pores. Among these, the first three components are considered to be separated by boundaries. Inner hydration products (including HD C-S-H and nanocrystalline phases) were set to grow inward from the original boundaries of cement particles in the model. For the outer hydration products, LD C-S-H forms through outward growth from the original cement particle boundaries, while outer crystalline phases deposit on the external surface. Together with the gel pores in the outward-growing hydration products, these form the outer hydration product layer.

After establishing the basic modeling principles, it was needed to quantify the volume changes before and after cement hydration. As mentioned earlier, GO acts as a nucleation site for cement hydration products, causing part of the LD C-S-H to transform into HD C-S-H. The former is part of the outer hydration product layer and is ion-transmissible, while the latter is part of the inner hydration product layer and is ion-non-transmissible. Thus, the transformation changes the compositional structure of hydration products and alters the volume fractions of the inner and outer hydration product layers. Using BSE imaging technology and Image J software (version 1.54g), the fraction that transformed from LD C-S-H to HD C-S-H was determined. According to the test results, after incorporating GO, the OPC-GO system produced 1.25 times the amount of HD C-S-H compared to the OPC system. Consequently, the volume fraction of the inner hydration layer became 1.5 times that without GO.

Therefore, for the cement paste of the OPC-GO system, the volume fractions of each phase can be calculated using Equations (5)–(12).

(5) $$V_{\mathrm{cem}}=\frac{V_{\mathrm{cem}}}{V_{\mathrm{cem}}+V_{\mathrm{water}}}$$

(6) $$V_{\mathrm{hydrated},\mathrm{cem}}=\alpha \cdot V_{\mathrm{cem}}$$

(7) $$V_{\mathrm{BJH}}=\varphi_{\mathrm{SCP}}^{P}$$

(8) $$V_{\mathrm{IP},\mathrm{cem}}=(1+0.15)\times V_{\mathrm{hydrated},\mathrm{cem}}$$

(9) $$V_{\mathrm{OP},\mathrm{cem}}=(k_{v}-1)\;\times \;V_{\mathrm{hydrated},\mathrm{cem}}-0.15\times V_{\mathrm{hydrated},\mathrm{cem}}$$

(10) $$V_{\mathrm{OC},\mathrm{cem}}=0.4\;\times \;(k_{v}-1)\;\times \;V_{\mathrm{hydrated},\mathrm{cem}}$$

(11) $$V_{\mathrm{OCSH},\mathrm{cem}}=0.6\;\times \;(k_{v}-1)\;\times \;V_{\mathrm{hydrated},\mathrm{cem}}-0.15\times V_{\mathrm{hydrated},\mathrm{cem}}$$

(12) $$V_{\mathrm{OCSHL},\mathrm{cem}}=V_{\mathrm{OCSH},\mathrm{cem}}+V_{\mathrm{BJH}}$$

where $V_{\mathrm{cem}},V_{\mathrm{hydrated},\mathrm{cem}},V_{\mathrm{BJH}},V_{\mathrm{IP},\mathrm{cem}},V_{\mathrm{OP},\mathrm{cem}},V_{\mathrm{OC},\mathrm{cem}},{\mathrm{and}\;V}_{\mathrm{OCSH},\mathrm{cem}}$ represent the volume fractions of cement, hydrated cement, gel pores, inner hydration products, outer hydration products, outer crystalline phases, and the outer C-S-H layer, respectively. $k_{v}$ represents the volume of a unit volume of cement after hydration.

**Figure 4:**
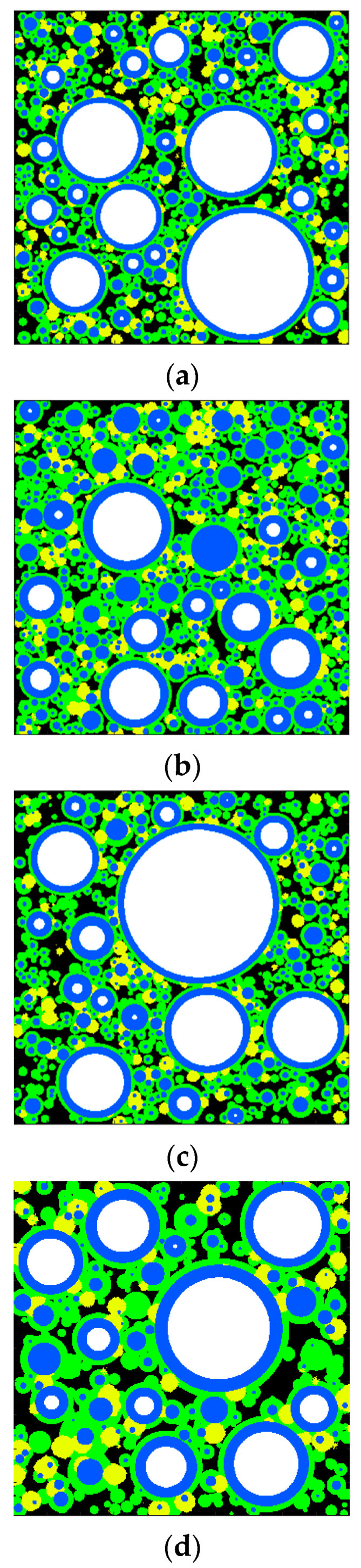
Two-dimensional slices of microstructure models for OPC and OPC-GO systems. (**a**) OPC: 7 days; (**b**) OPC: 56 days; (**c**) OPC-GO: 7 days; (**d**) OPC-GO: 56 days. The white region represents unhydrated cement particles, blue represents the inner hydration product layer, green represents the outer C-S-H layer, yellow represents the outer large crystalline phase, and black represents pores.

Based on the volume changes, the modeling of the cement paste microstructure was conducted for OPC and OPC-GO systems at varied ages. Figure 4 shows the 2D images of the microstructure models for the OPC and OPC-GO systems.

**Figure 5:**
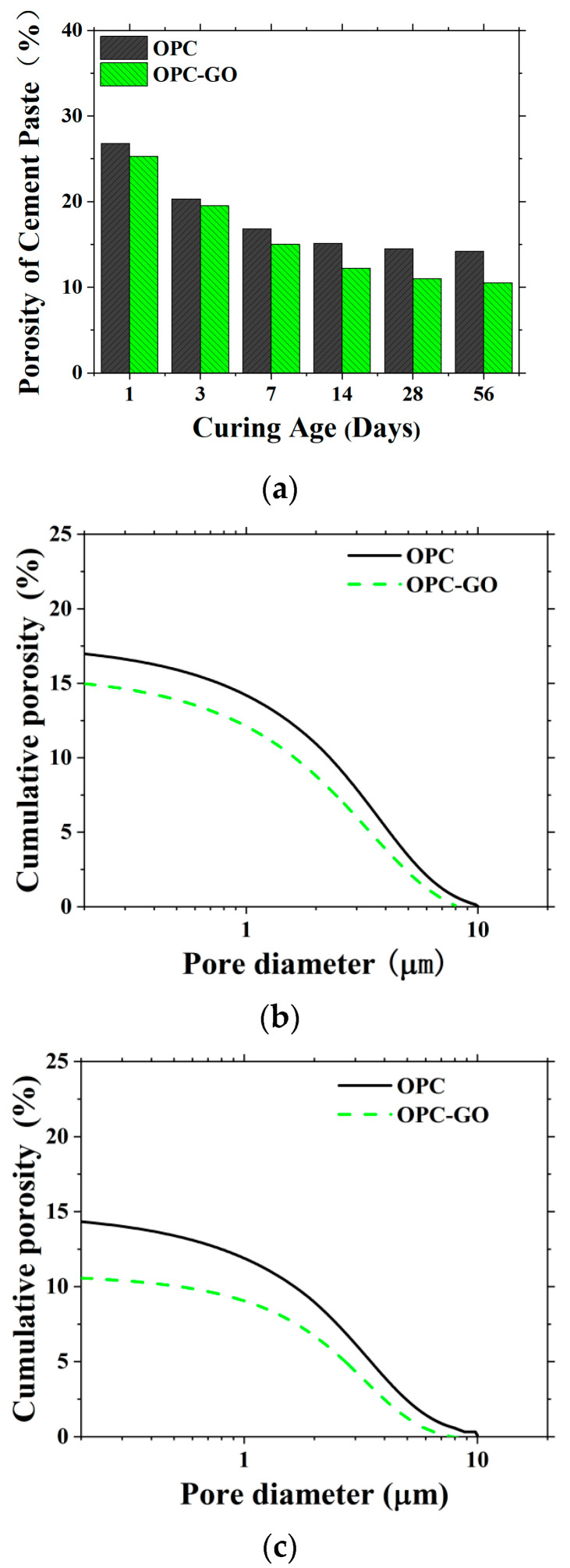
The calculated porosity of cement pastes in mortar at varied ages (**a**). Calculated cumulative porosity of OPC and OPC-GO systems at the age of 7 days (**b**) and 56 days (**c**).

Figure 5 shows the cement paste porosity at different ages and the cumulative pore volume distribution at 7 and 56 days for both OPC and OPC-GO systems. It can be seen that GO addition reduced both the porosity and the maximum pore diameter of the cement matrix at both early and later ages. This indicates that at the microscale, GO addition improves the density of cement paste and optimizes the pore structure.

**Figure 6:**
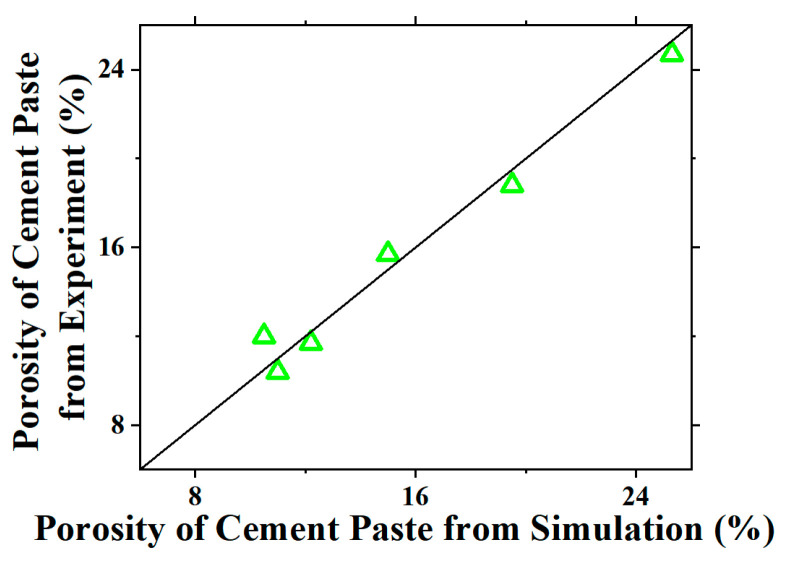
The comparison between the measured porosity and the one calculated from the microstructure model for OPC-GO paste.

To verify the microstructure model, BSE images were obtained for the OPC-GO system, and Image J software was used for image processing to obtain the porosity. The comparison between the measured porosity and the one calculated from the model is shown in Figure 6. It can be seen that the data points are all distributed near the diagonal line, proving the accuracy of the simulation for the OPC-GO system.

#### 4.2.3. The Microstructure Modeling of the ITZ in Mortar at the Microscale

**Figure 7:**
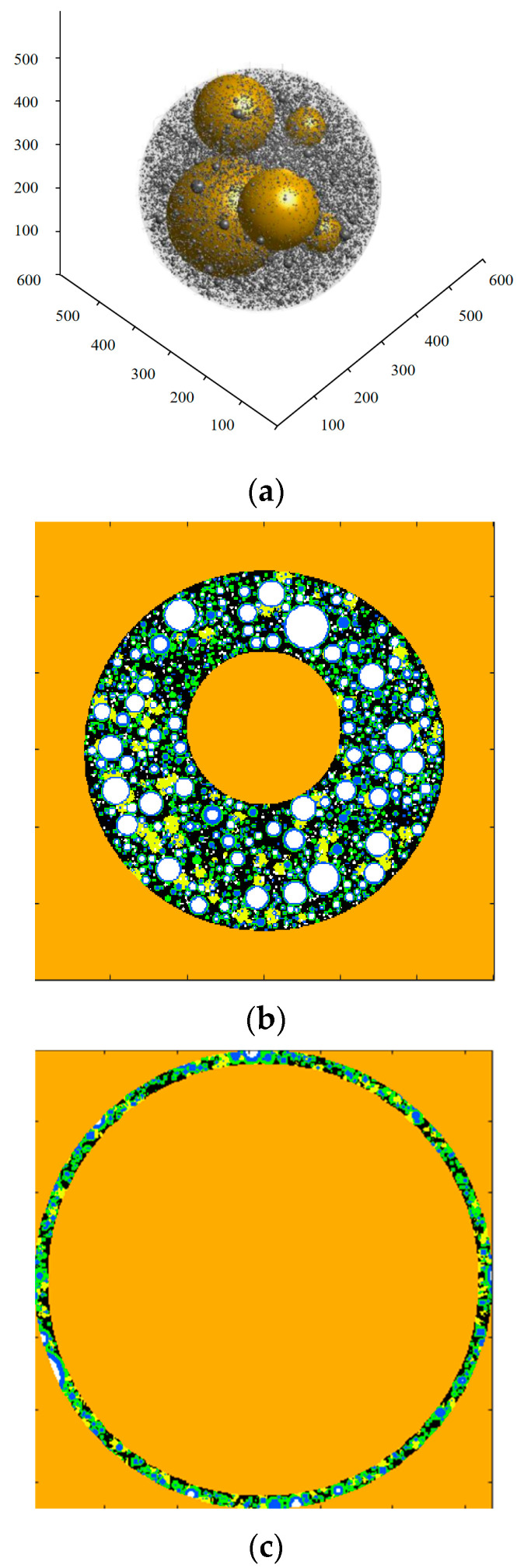
Modeling of the ITZ using the multi-aggregate method. (**a**) 3D image of the multi-aggregate model. (**b**) 2D slide obtained from the multi-aggregate model. (**c**) 2D slice of the ITZ microstructure cut out from the multi-aggregate model. In (**b**,**c**), the white region represents unhydrated cement particles, blue represents the inner hydration product layer, green represents the outer C-S-H layer, yellow represents the outer large crystalline phase, and black represents pores.

While establishing the ITZ model for the OPC-GO system, considering that the existence of the ITZ depends on the presence of aggregates, the ITZ was not treated as an independent entity but was modeled using a probability method [46]. Therefore, a multi-aggregate method [47] was adopted to establish a multi-aggregate model from which the ITZ could be extracted. Figure 7 illustrates the ITZ modeling process using the multi-aggregate method. The details are described as follows.

In the space where aggregates and cement particles are situated (Figure 7a), cement particles undergo hydration to form hydration products (Figure 7b). For the ITZ, although external nuclei for growth are randomly distributed as in the cement matrix, their probability of existence varies for different regions due to the presence of aggregate phases. The probability is lowest on the surface of hydrated cement particles, higher in large capillary pores, and highest on aggregate surfaces. Then, around any aggregate, a thin shell with an ITZ thickness value of *t*_ITZ_ can be cut out, and this thin shell is the ITZ microstructure model (Figure 7c).

During the process of establishing the ITZ microstructure model, the input value required by the program was the hydration degree of cement, which had been calculated in the hydration kinetics model established in Section 4.1.2. According to the obtained ITZ thickness *t*_ITZ_, the ITZ porosity can also by calculated with the ITZ model.

**Figure 8:**
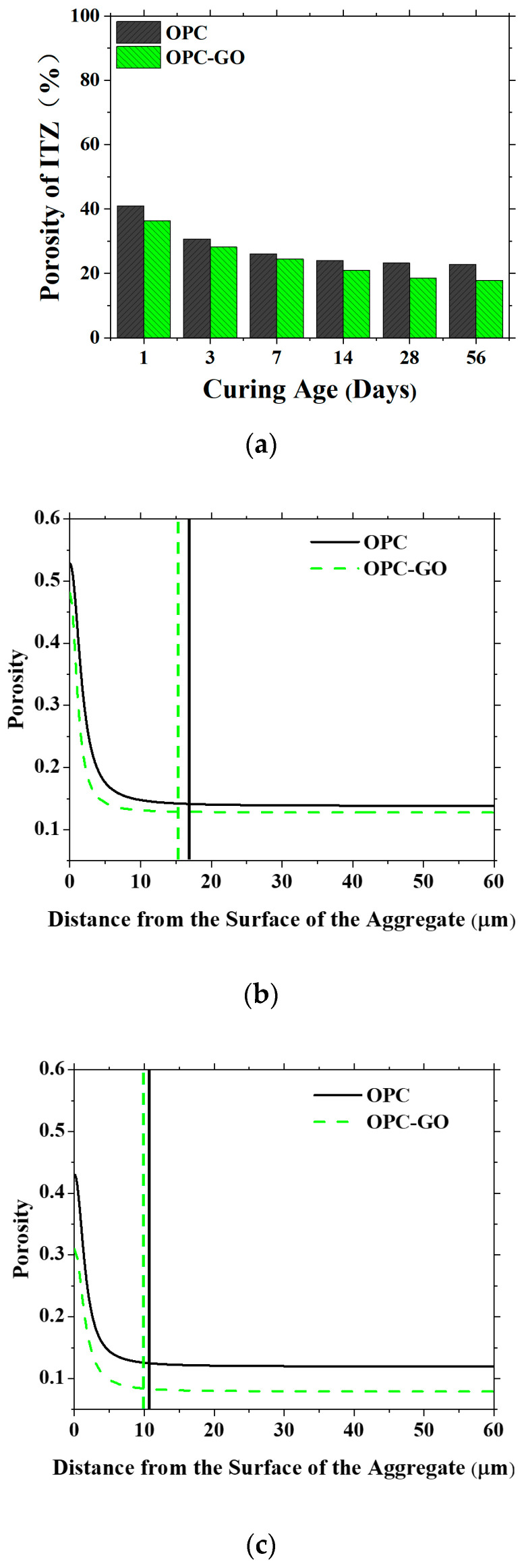
(**a**) Porosity of the ITZ in OPC and OPC-GO systems at varied ages. (**b**,**c**) The pore distribution of the ITZ in the two systems at the age of 7 and 56 days, respectively.

The ITZ model was established for OPC and OPC-GO systems at varied ages, and the ITZ porosity and the pore distribution for the two systems are shown in Figure 8. The addition of GO reduced the ITZ porosity as well as the ITZ thickness at both early and later ages, which is consistent with the experimental findings in the literature [48]. Therefore, GO addition could significantly improve the microstructure of the ITZ in cementitious materials.

#### 4.2.4. The Microstructure Modeling of Cement Mortar at the Mesoscale

**Figure 9:**
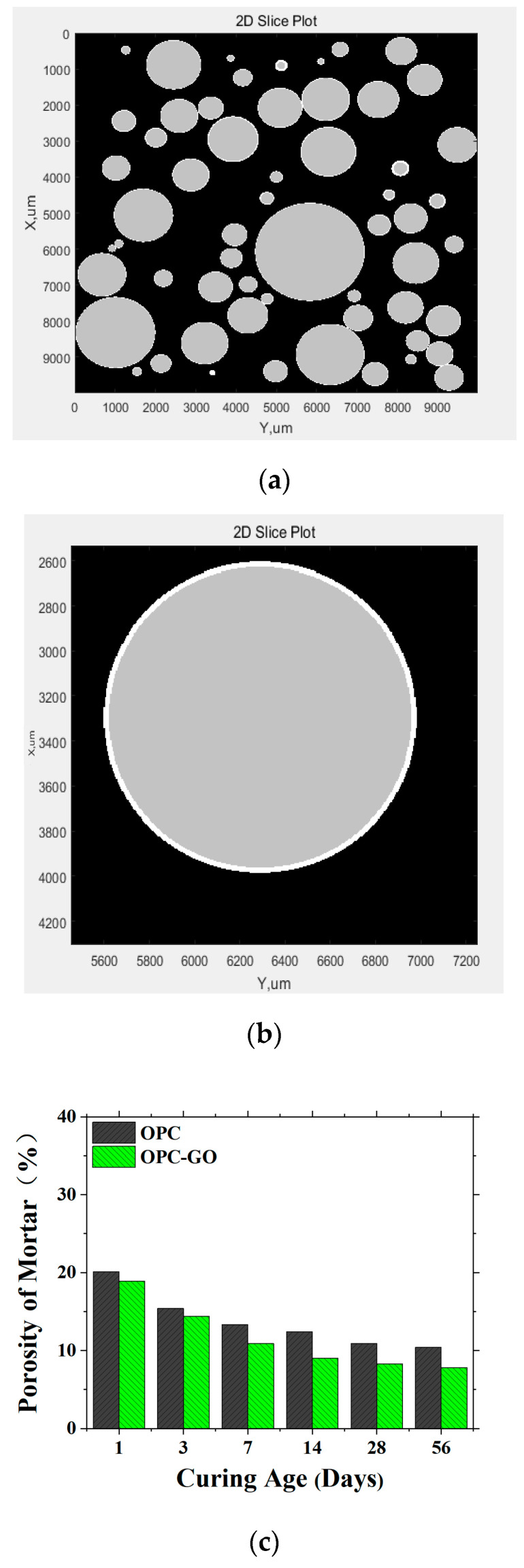
The microstructure model for the mortar ((**a**) 2D slide; (**b**) enlarged aggregate). The gray areas represent aggregate particles, white areas represent the ITZ, and black areas represent the cement matrix. (**c**) Porosity for OPC and OPC-GO mortars at varied ages.

To determine the microstructure of the OPC-GO mortar, the mortar can be considered as a three-phase body consisting of the cement matrix, aggregate particles, and the ITZ between the aggregate particles and cement matrix, thereby establishing a cement mortar microstructure model. The modeling process is as follows. First, generate a space with dimensions of 10 mm × 10 mm × 10 mm with a spatial resolution of 5 μm. Then, based on the sand particle size distribution, put aggregates into the space until reaching the preset aggregate volume fraction V_agg_. Next, using the *t*_ITZ_ obtained from the model at the microscale, generate an ITZ shell outward from each aggregate, with the remaining space still being the bulk cement paste. In this modeling process, the other required inputs are the porosity of the cement matrix and the ITZ obtained from the model at the microscale. After modeling, the microstructure model of cement mortar and the mortar porosity are obtained (Figure 9a,b).

The microstructure model was established for both OPC and OPC-GO mortar and the calculated porosity of the mortars can be found in Figure 9c. It can be seen that whether in the early or later stages of hydration, GO addition can reduce the cement mortar porosity. This indicates that at the mesoscale, GO addition has a positive effect on the microstructure of the mortar.

**Figure 10:**
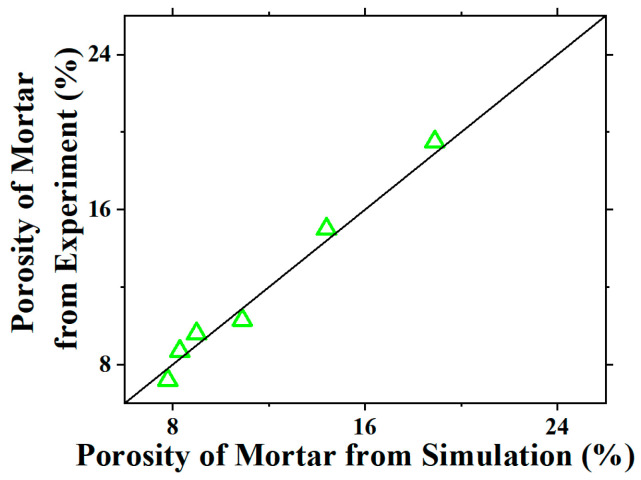
The comparison between the measured porosity and the one calculated from the microstructure model for OPC-GO mortar.

To validate the microstructure model for the OPC-GO mortar, BSE images were taken and analyzed to obtain the porosity for the mortar. The comparison between the measured porosity and the one calculated from the model is shown in Figure 10. It can be seen that all data points are distributed around the diagonal line, confirming the effectiveness of the microstructure model for OPC-GO mortar.

**Figure 11:**
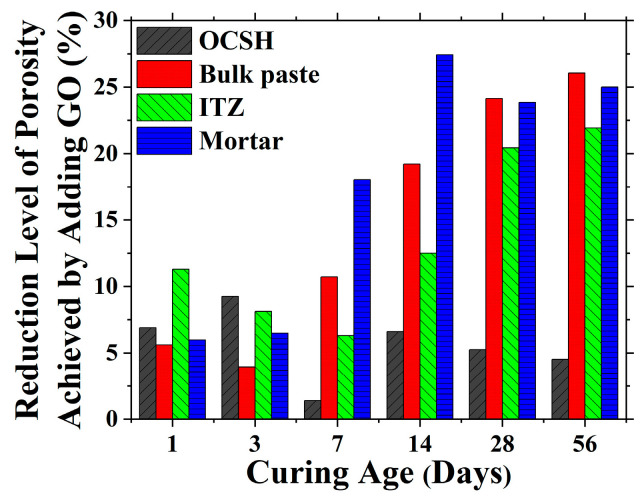
Comparison of reduction level of porosity achieved by adding GO for OCSH, bulk paste, ITZ, and mortar with different curing ages.

To evaluate the influence of GO incorporation on products at different scales, the reduction in porosity for the outer calcium silicate hydrate (OCSH), bulk paste, interfacial transition zone (ITZ), and mortar structures across various ages is presented in Figure 11 and Table 4. The results demonstrate that GO incorporation had a relatively minor effect on the OCSH porosity (maximum reduction of 6.9%), while exhibiting substantial influence on the porosity of the bulk paste, the ITZ, and consequently, the mortar (maximum reduction of 27%). Furthermore, GO demonstrated superior porosity control at later ages compared to early ages. However, this observation may be attributed to the decreased porosity of the OPC used for normalization. Therefore, a comprehensive analysis of GO’s influence on transport properties is necessary before drawing definitive conclusions.

### 4.3. The Modeling of the Transport Properties of Cementitious Materials with GO

**Figure 12:**
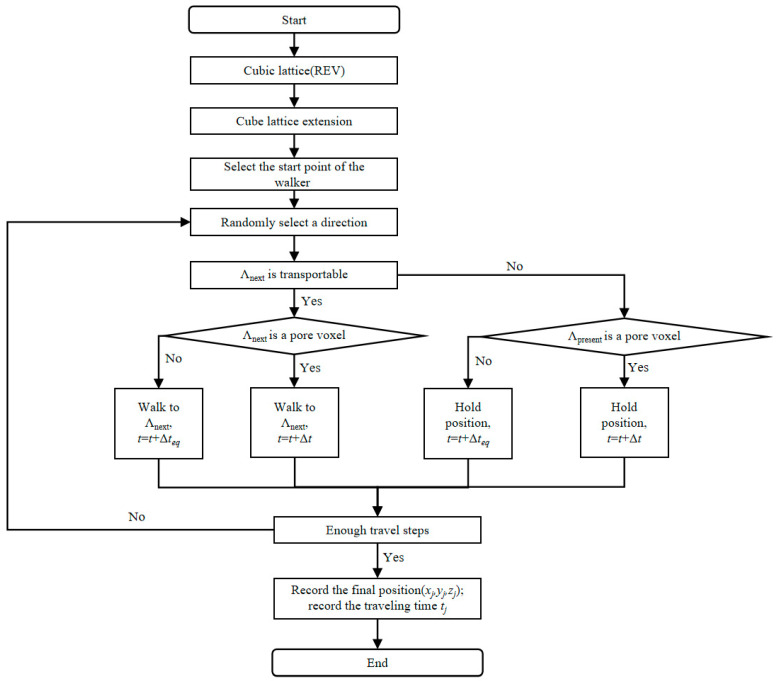
Flow diagram of the random walk algorithm implementation for the porous media transport simulation (reprinted from Ref. [33]). In the diagram, *t* represents the traveling time of the walker. The time increment Δ*t* is fixed at unity to maintain consistency with free-space diffusion simulations. Δ*t*_eq_ = τ_D_Δ*t*.

Based on the microstructure models established at multiple scales, transport property simulations could be conducted at these scales for the OPC-GO system using a random walker algorithm. The random walker algorithm references the classic random walk algorithm of an ant, using walkers to represent transportable substances (such as harmful ions) to simulate their random diffusion in cementitious materials [33]. A detailed flowchart is shown in Figure 12. This algorithm has been successfully applied to transport property simulations of concretes, and its accuracy and adaptability have been experimentally verified.

The transport property simulation process for the OPC-GO system required us to identify the ion-transportable phases in the established sub-micron, micron, and mesoscopic microstructure models. At the sub-microscale, the transportable phases are gel pores and low-density C-S-H. The diffusion tortuosity $\tau_{D}^{\mathrm{SCP}}$ of gel pores is 1, while the diffusion tortuosity of LD C-S-H $\tau_{D}^{\mathrm{LDCSH}}$ can be set to 21.76 [33]. The two parameters are input parameters for the program. After simulation, the program outputs the diffusion tortuosity $\tau_{D}^{\mathrm{OCSHL}}$ of the outer C-S-H layer.

At the microscale, the transportable phases are the outer C-S-H layer and large capillary pores, while the non-transportable phases are unhydrated cement, inner hydration products, and crystalline phases. The diffusion tortuosity of large capillary pores is 1, while the diffusion tortuosity of the outer C-S-H layer was obtained from the sub-microscale simulation. The two values are input into the program for simulation. After simulation, the output parameters are the diffusion tortuosities $\tau_{D}^{P}\;and\;\tau_{D}^{\mathrm{ITZ}}$ for the cement paste matrix and ITZ, respectively.

At the mesoscale, the transportable phases include the cement paste matrix and ITZ. In the simulation, $\tau_{D}^{P}$ and $\tau_{D}^{\mathrm{ITZ}}$ obtained from the microscale simulation are input into the program. After simulation, the output value is the diffusion tortuosity τ_D_ of the cement mortar, which is the final output result. τ_D_ is a crucial parameter to characterize the diffusion performance of ions. A larger value of τ_D_ indicates greater resistance to ion diffusion in cementitious materials, meaning better resistance to harmful ion penetration.

**Figure 13:**
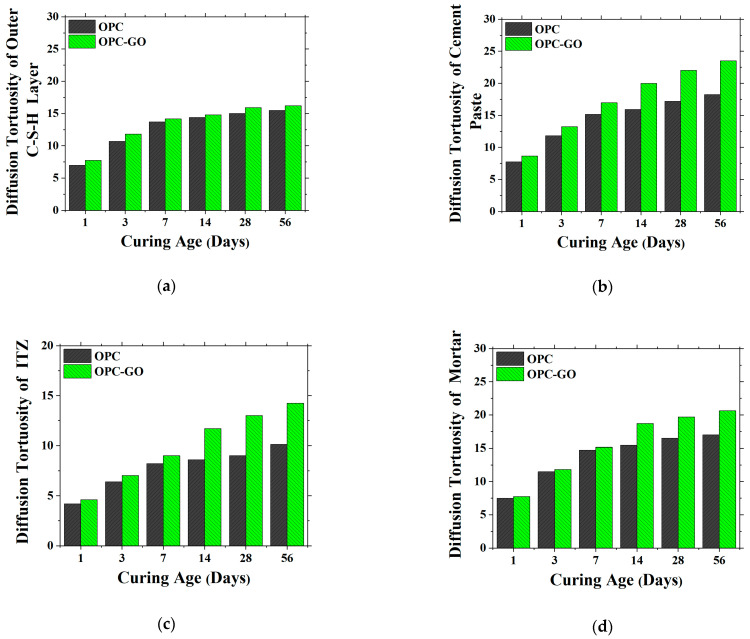
The diffusion tortuosity $\tau_{D}^{\mathrm{OCSHL}}$ of the outer C-S-H layer at the sub-microscale (**a**), the $\tau_{D}^{P}$ and $\tau_{D}^{\mathrm{ITZ}}$ of the cement paste matrix (**b**) and ITZ (**c**) at the microscale, and the τ_D_ of mortar (**d**) for OPC and OPC-GO systems.

Figure 13a shows the diffusion tortuosity $\tau_{D}^{\mathrm{OCSHL}}$ of the outer C-S-H layer in the OPC and OPC-GO systems at the sub-microscale. It could be found that at all ages, GO addition increased the diffusion tortuosity of the outer C-S-H layer. This was mainly because GO addition reduced the porosity of the outer C-S-H layer. This indicates that at the sub-microscale, GO addition improved the resistance to penetration by harmful ions in cementitious materials.

Figure 13b,c show the diffusion tortuosity of cement paste and the ITZ for the OPC and OPC-GO systems at the microscale. The simulation results indicate that the presence of GO also led to an increase in the diffusion tortuosity of both the cement paste matrix and ITZ at all the curing ages, with a more significant enhancement observed at later ages. This indicates that at the microscale, GO addition can improve the ion penetration resistance of cementitious materials. The reason for this is due to the lower porosity with GO on the one hand and the LD C-S-H transforming into non-transportable HD C-S-H on the other hand.

Moreover, it was noticed that the diffusion tortuosity of the ITZ was smaller than that of the cement paste matrix, reflecting the weaker ion penetration resistance property of the ITZ. This also proved the necessity of simulating the transport properties of ions separately for cement paste and the ITZ.

Figure 13d shows the diffusion tortuosity τ_D_ of mortar in the OPC and OPC-GO systems at the mesoscale. It could be found that at all ages, GO addition increased the diffusion tortuosity of cement mortar as expected due to the characteristics presented at the sub-micron scale and microscale.

In summary, the effect of GO incorporation on the ion penetration resistance of cementitious materials was analyzed at three scales: sub-micron, micron, and mesoscopic. At all three scales, GO could improve the ion penetration resistance of cementitious materials due to the following reasons. GO acts as a physical filler, provides nucleation sites, and serves as a reactant. These mechanisms collectively densify the microstructure and reduce porosity through physical filling effects and enhanced cement hydration, thereby impeding ionic transport through the pore network. Consequently, the diffusion tortuosity in the transportable phases of hydration products increased at all simulation scales, making ion transport more difficult.

**Figure 14:**
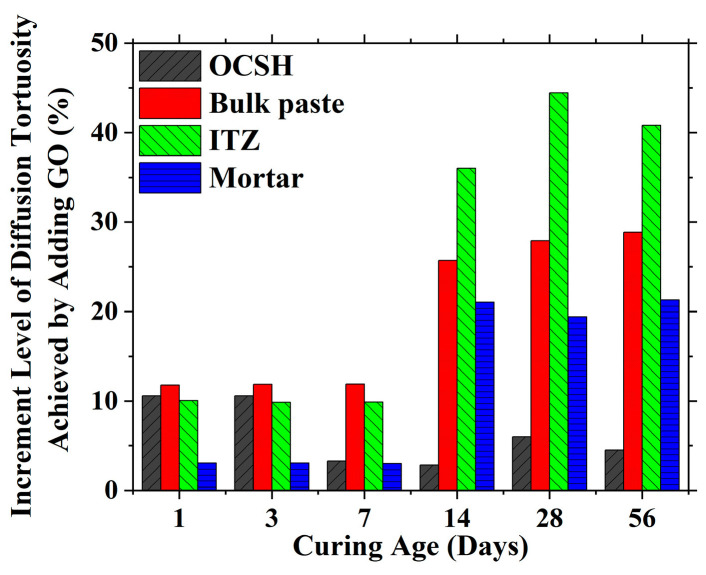
Comparison of increment level of diffusion tortuosity achieved by adding GO for OCSH, bulk paste, ITZ, and mortar with different curing ages.

Figure 14 and Table 5 compare the influence of GO incorporation on the ionic diffusion tortuosity in the outer C-S-H (OCSH), bulk paste, ITZ, and mortar. The results indicate that GO incorporation had a minimal effect on the OCSH tortuosity (increase of less than 10%), while exhibiting a significantly greater impact on the bulk paste, ITZ, and mortar (maximum increase of 44%). This suggests that GO primarily enhances the transport properties of cementitious materials by modifying relatively larger pore structures compared with the nano-sized pores within the cement paste and ITZ. Moreover, it should be noticed that GO demonstrated a more pronounced influence on the diffusion tortuosity at later ages (after 14 days) compared to early ages, consistent with its effect on the porosity. This observation indicates the more effective functionality of GO to improve the transport properties of cementitious materials, as well as the durability, at later ages.

Despite the successful establishment of a multi-scale model considering the microscopic to macroscopic levels that yields results consistent with experimental observations, certain limitations of the current model should be acknowledged.

(1) The present model represents a simplified mortar system. While its extension to concrete simulation would primarily require the incorporation of coarse aggregates at the mesoscale and the corresponding spatial expansion, such modifications would significantly increase the computational demands, necessitating enhanced computing capabilities.

(2) The model is based on the assumption that GO particles are uniformly distributed within the cementitious matrix without agglomeration, thereby enabling each GO particle to function at maximum efficiency. However, in practice, as the GO content increases, dispersion challenges inevitably arise due to its nanoscale characteristics. GO agglomerates not only compromise the performance enhancement but also generate additional porosity through spatial hindrance and dilution effects, ultimately leading to deteriorated properties of the cementitious materials. This phenomenon aligns with previous studies that identified an optimal GO dosage threshold [19]. To utilize the model for GO content optimization, further development is required to incorporate agglomeration thresholds and the morphological characteristics of GO clusters, which constitutes our future research direction.

(3) The diffusion tortuosity obtained from numerical simulations can be utilized to predict multiple transport-related properties of cementitious materials, including the electrical conductivity, chloride ion diffusion (penetration) coefficients, and intrinsic permeability. Nevertheless, experimental validation and appropriate calibration procedures may be required to ensure accurate quantitative predictions.

## 5. Conclusions

By combining hydration modeling, multi-scale microstructure modeling, and random walker algorithms, a transport model for cementitious materials with a GO admixture was established to predict the transport behavior of hazardous ions within cementitious materials.

The hydration kinetics model of the OPC-GO composite system revealed that the addition of GO reduces the duration of the induction period and increases the rate of hydration development after the induction period ends. The multi-scale microstructure model of the OPC-GO system also showed that GO addition reduces the porosity of cementitious materials at all simulation scales during both early and later ages. Furthermore, at the microscale, it enhanced the pore structure of the cement matrix and ITZ by decreasing the size of large pores and increasing the number of small pores. The transport simulation of the OPC-GO system revealed that GO could enhance the resistance to hazardous ion ingress of cementitious materials at all three simulation scales.

The simulation results provide a reference for improving the durability of cementitious materials in marine environments and promoting the applications of GO-related nanomaterials. In fact, this model’s establishment is not only applicable to simulating interactions between GO and cementitious materials, but also can be extended to other additive systems through parameter adjustments.

## Figures and Tables

**Table 1:** Mineral composition and physical parameters of OPC.

Mineral Composition	Mass Fraction (%)
$C_{3}S$	58.38
$C_{2}S$	15.22
$C_{3}A$	7.57
$C_{4}\mathrm{AF}$	12.13
$C\overset{-}{S}H_{2}$	4.62
Specific density	3.12

**Table 2:** Fitting parameters in Equation (1) for the OPC and OPC-GO systems.

Specimen Identification	$\alpha_{u}$	τ	β	$R^{2}$
OPC	0.766	18.04	0.77	0.9954
OPC-GO	0.78	16.99	0.88	0.9844

**Table 3:** Porosity of gel pores $\left(\varphi_{\mathrm{SCP}}^{P}\right)$ in OPC and OPC-GO systems at varied ages (%).

Specimen Identification	1 Day	3 Days	7 Days	14 Days	28 Days	56 Days
OPC	17	12.1	9.7	9	8.3	8
OPC-GO	15.4	11.2	9.3	8.7	8	7.7

**Table 4:** Comparison of reduction level of porosity achieved by adding GO for OCSH, bulk paste, ITZ, and mortar with different curing ages (%).

Phase Identification	1 Day	3 Days	7 Days	14 Days	28 Days	56 Days
OCSH	6.90	9.25	1.39	6.61	5.25	4.51
Bulk paste	5.60	3.94	10.71	19.21	24.14	26.06
ITZ	11.29	8.11	6.30	12.50	20.43	21.93
Mortar	5.97	6.49	18.05	27.42	23.85	25.00

**Table 5:** Comparison of increment level of diffusion tortuosity achieved by adding GO for OCSH, bulk paste, ITZ, and mortar with different curing ages (%).

Phase Identification	1 Day	3 Days	7 Days	14 Days	28 Days	56 Days
OCSH	10.59	10.57	3.28	2.85	6.00	4.52
Bulk paste	11.77	11.84	11.88	25.71	27.91	28.85
ITZ	10.05	9.84	9.88	36.00	44.44	40.81
Mortar	3.07	3.05	2.99	21.04	19.39	21.29

## Data Availability

Data are contained within the article.
